# Extraction of Carnosic Acid and Carnosol from Sage (*Salvia officinalis* L.) Leaves by Supercritical Fluid Extraction and Their Antioxidant and Antibacterial Activity

**DOI:** 10.3390/plants8010016

**Published:** 2019-01-09

**Authors:** Valentina Pavić, Martina Jakovljević, Maja Molnar, Stela Jokić

**Affiliations:** 1 Department of Biology, Josip Juraj Strossmayer University of Osijek, Cara Hadrijana 8/A, 31000 Osijek, Croatia; 2 Faculty of Food Technology Osijek, Josip Juraj Strossmayer University of Osijek, Franje Kuhača 20, 31000 Osijek, Croatia; mjakovljevic@ptfos.hr (M.J.); maja.molnar@ptfos.hr (M.M.); stela.jokic@ptfos.hr (S.J.)

**Keywords:** *Salvia officinalis* L., supercritical CO_2_ extraction, carnosic acid, carnosol, optimization, antibacterial activity, antioxidant activity

## Abstract

Sage (*Salvia officinalis* L.) is a good source of antioxidant compounds, carnosic acid and carnosol being the prominent ones. Both are soluble in CO_2_, and our goal was to investigate the application of supercritical CO_2_ extraction to obtain sage extracts rich in these compounds. The effect of pressure, temperature, and CO_2_ flow rate on the carnosic acid and carnosol yield was studied. These variables were optimized by response surface methodology (RSM). The pressure significantly affected carnosol extraction, while the extraction of carnosic acid was affected by the pressure, temperature, and CO_2_ flow rate. Carnosic acid content varied from 0.29–120.0 µg mg^−1^, and carnosol content from 0.46–65.5 µg mg^−1^. The optimal conditions according to RSM were a pressure of 29.5 MPa, a temperature of 49.1 °C, and a CO_2_ flow rate of 3 kg h^−1^, and the sage extract yield was calculated to be 6.54%, carnosic acid content 105 µg mg^−1^, and carnosol content 56.3 µg mg^−1^. The antioxidant activities of the sage extracts were evaluated by the scavenging activities of 2,2-diphenyl-1-picrylhydrazyl (DPPH). Sage extract obtained at 30 MPa and 40 °C with 2 kg h^−1^ CO_2_ flow rate with a carnosic acid content of 72 µg mg^−1^ and carnosol content of 55 µg mg^−1^ exhibited the highest antioxidant activity (80.0 ± 0.68%) amongst the investigated supercritical fluid extracts at 25 µg mL^−1^ concentration. The antimicrobial properties of extracts were tested on four bacterial strains: *Escherichia coli*, *Pseudomonas aeruginosa*, *Bacillus subtilis*, and *Staphylococcus aureus*. The extract with a carnosic acid content of 116 µg mg^−1^ and a carnosol content of 60.6 µg mg^−1^ was found to be the most potent agent against *B. subtilis*.

## 1. Introduction

Sage (*Salvia officinalis* L.), a member of the Lamiaceae family, is an aromatic medicinal plant often used in culinary preparations and in folk medicine for various health conditions, such as fever and sweating, rheumatism, bronchitis, and mental and nervous disorders [1,2]. Numerous studies have shown a positive effect of various sage extracts on human health (e.g., tea, essential oils, ethanolic extracts, etc.). The complex composition of sage extracts, considering bioactive compounds such as terpenes (monoterpenes, diterpenes, triterpenes) and phenolic compounds, is the reason for their biological activities and health effects [3,4,5,6].

The most prevalent phenolic components in sage extracts are phenolic acids (caffeic, vanillic, ferulic, and rosmarinic acids) and flavonoids (lutein, apigenin, and quercetin) [7,8], while the most abundant components with antioxidant activity are primary diterpenes such as carnosic acid, carnosol, and methyl carnosate [9,10], followed by flavonoids and other phenols [7].

Carnosic acid is a phenolic diterpene belonging to the class of the secondary plant metabolites called terpenoids, isoprenoids, or terpenes [11]. Carnosol (picrosalvin) is an ortho-diphenolic diterpene and an oxidative derivative of carnosic acid [12], formed in the presence of oxygen, after plant harvest and during the leaf drying process [13,14]. If exposure to air occurs during the extraction process, phenolic diterpenes with a lactone structure are also formed, such as rosmanol, epirosmanol, and 7-methyl-epirosmanol [15].

Carnosic acid is mostly present in the aerial parts of the plant, and the content of carnosic acid and carnosol in sage leaves increases with senescence [16]. Dried rosemary or sage leaves can contain between 0.1% and 7% carnosic acid, depending on the species, variety, plant growth conditions, sample treatment, and type of extract preparation [17]. In different studies [18,19], sage leaves contained 2.12 mg g^−1^ DW and 1.34 mg g^−1^ DW carnosic acid and carnosol. Carnosic acid was proven to be very efficient in the prevention of fish oil oxidation and its antioxidant activity was shown to be stronger than that of vitamin E [20], while Guitard et al. [21] also found that carnosic acid is very efficient in the preservation of omega-3 oils. Beside their antioxidant activity, the antibacterial activity of carnosic acid and carnosol against both gram-positive and gram-negative bacteria was also confirmed [22,23,24]. Furthermore, Horiuchi et al. [25] found that a crude extract of sage reduced the minimum inhibitory concentrations (MICs) of aminoglycosides in vancomycin-resistant enterococci. Carnosic acid and carnosol, as important antioxidants in plants, have been suggested to account for over 90% of the antioxidant properties of rosemary extract [26]. These are the main reasons to investigate these two compounds in sage extracts. 

So far, various extraction techniques have been employed to obtain sage extracts rich in bioactive components, hydrodistillation [27,28,29,30,31], solid–liquid extraction involving ultrasonic and microwave application [8,28,32,33,34], and supercritical fluid extraction (SFE) [35,36,37,38]. 

SFE has attracted attention in recent years in the process of separation as a highly desirable “green“ solvent, since it is a non-toxic, non-flammable, odorless, tasteless, inexpensive, readily available in large quantities, and environmentally friendly solvent [39]. Since a large number of parameters can influence the SFE process, it is important to perform screening for the detection of the most influential ones on the investigated responses. For this purpose, response surface methodology (RSM) is appropriate, because of the possibility of finding optimal levels of factor in the SFE process [40]. RSM, which was originally described by Box and Wilson [41], proved to be very useful in modeling and analyzing problems with the influence of several variables on the response.

In our latest paper [42], we investigated the SFE of sage leaves for selected components, such as oxygenated monoterpenes, α-humulene, viridiflorol, and manool. In this work, on the other hand, we focused on the carnosic acid and carnosol content of sage SFE extracts in order to obtain sage extracts enriched in these phenolic diterpenes.

A literature search revealed that there is no data available on SFE parameter optimization in order to achieve the optimal content of carnosic acid and carnosol in sage extracts, as it is presented in our research. Both Babović et al. [43] and Glisic et al. [44] have performed SFE of sage leaves, and there are also some other researches on the SFE of sage [36,37,38]; however, those were mainly focused on yield, relative percentage of volatile compounds, or the determination of the extract’s composition at selected extraction conditions.

Considering all the above, the objectives of this study were focused on investigating the influence of various SFE parameters (pressure, temperature, and CO_2_ flow rate) on: (1) the content of carnosic acid and carnosol in sage extracts analyzed by HPLC; (2) defining the optimal extraction conditions by RSM for the desired antioxidant components (carnosic acid and carnosol); and (3) defining the optimal extraction conditions for antioxidant and antibacterial activity.

## 2. Materials and Methods

### 2.1. Chemicals

Commercial CO_2_ used for SFE was 99.97% (*w*/*w*) pure (Messer, Osijek, Croatia). 2,2-Diphenyl-1-picrylhydrazyl (DPPH), ascorbic acid (AA), gallic acid, carnosic acid, and carnosol were purchased from the Sigma Chemical Co. (St. Louis, MO, USA). Other solvents were obtained from J.T. Baker (Radnor, PA, USA).

### 2.2. Plant Material

Sage leaves (*S. officinalis* L.) were obtained in spring 2016 from herbal pharmacy Vextra d.o.o. (Mostar, Bosnia and Herzegovina). In the sage leaves, moisture content (12.42% with an S.D. of 0.06) and the particle size of the ground leaves was determined as described previously [42]. Each measurement was performed in triplicate.

### 2.3. Supercritical Fluid Extraction and Experimental Design

An SFE system, explained in detail in [45], was used for extraction experiments. An extraction procedure of the sage leaves was also explained in detail previously [42]. Briefly, 50.0 g of the ground sage leaves was extracted in each experiment. Box-Behnken design (BBD) was chosen to create different extraction experiments (Table 1 and Table 2) and *Design-Expert*^®^ commercial software (ver. 9, Stat-Ease Inc., Minneapolis, MN, USA) was used for data analysis.

### 2.4. Chemical Characterization of the Extracts

HPLC analysis of carnosol and carnosic acid was performed on a Varian ProStar system (Varian Analytical Instruments, Palo Alto, CA, USA), with a Varian ProStar 230 Solvent Delivery Module, a ProStar 500 Column Valve Module, and a ProStar 330 Photodiode Array detector. Chromatographic separation was performed on a COSMOSIL 5C18-MA-II (Nacalai Tesque, Inc., Kyoto, Japan) 250 mm-long column with an internal diameter of 4.6 mm.

Separation of analyzed compounds was performed by isocratic elution for 40 min, where acetonitrile was used as phase A and 0.1% H_3_PO_4_ (in millipore water) as phase B, with a 60:40 ratio of A:B. The flow rate was 1.0 mL min^−1^, the injection volume was 20 μL, the UV detection wavelength was 230 nm, and the analysis was performed at room temperature.

Standard stock solutions for carnosic acid and carnosol were prepared in a solvent and calibration was obtained at eight concentrations (concentration range 10.0, 20.0, 30.0, 50.0, 75.0, 100.0, 150.0, and 200.0 mg L^−1^). The linearity of the calibration curve was confirmed by *R*^2^ = 0.9997 for carnosic acid and *R*^2^ = 0.9997 for carnosol. For carnosic acid, the limit of detection (LOD) was 0.082 mg L^−1^, the limit of quantification (LOQ) was 0.273 mg L^−1^, and the compound retention time was 20.3 min. For carnosol, the LOD was 0.103 mg L^−1^, the LOQ was 0.344 mg L^−1^, and the compound retention time was 13.4 min.

### 2.5. Determination of Total Phenolics Content

The total phenolics contents of SFE sage leaf extracts were determined by a spectrophotometric method with Folin–Ciocalteu reagent, calibrated against gallic acid [46]. The results were calculated according to the calibration curves for gallic acid, derived from triplicate analyses and expressed as milligrams of gallic acid equivalents (GAE) per gram of dry mater.

### 2.6. 2,2-Diphenyl-1-picrylhydrazyl (DPPH) Radical Scavenging Activity

Total antioxidant activities of SFE sage leaf extracts were determined using the DPPH radical scavenging assay described earlier [47]. The plant extracts were dissolved in methanol (25 μg mL^−1^) and mixed with 0.2 mM DPPH radical solution. Ascorbic acid (AA) was used as a reference compound. All measurements were done in triplicate. The absorbance was measured at 517 nm, and DPPH scavenging activity was determined using Equation (1):

DPPH activity = (A_b_ + A_s_) − A_m_)/A_b_ × 100 (1)

where A_b_ is the absorbance of 0.1 mM DPPH radical solution at λ = 517 nm, A_s_ is the absorbance of 0.1 mM extraction solution at λ = 517 nm, and A_m_ is the absorbance of 0.1 mM solution mixture of tested extracts and DPPH radical at 517 nm.

### 2.7. Antibacterial Susceptibility Testing

#### 2.7.1. Microorganisms and Growth Conditions

Two gram-positive *Bacillus subtilis* and *Staphylococcus aureus*, and two gram-negative *Escherichia coli* and *Pseudomonas aeruginosa*, were investigated. The four bacteria were isolates from various clinical specimens obtained from the Microbiology Service of the Public Health Institute of Osijek-Baranja County, Croatia. *B. subtilis* and *E. coli* were selected as two popular laboratory model organisms representing gram-positive and gram-negative bacteria, respectively. *S. aureus* and *P. aeruginosa* were selected as human pathogens representing gram-positive and gram-negative bacteria, respectively. Working cultures were prepared from subcultures and grown overnight in Muller Hinton Broth (MHB) (Fluka, BioChemica, Germany) under optimal conditions for each microorganism. The antibacterial agent gentamicin (BioChemica, Germany) was dissolved in distilled water.

#### 2.7.2. Minimum Inhibitory Concentration (MIC), Growth Inhibition and 50% Growth Reduction (IC_50_)

MIC and 50% growth reduction (IC_50_) values were determined by a modified broth microdilution method [48] as described in our previous work [49]. The MIC and IC_50_ were defined as the lowest concentration of the extract which completely inhibited the growth of a particular microorganism, and the concentration which inhibited 50% of growth, respectively. Assays were performed with sterile TPP 96-well plates (TPP Techno Plastic Products AG Trasadingen, Switzerland) in a final volume of 200 μL. A total of 100 μL of midlogarithmic-phase bacterial cultures (5 × 10^5^ CFU mL^−1^) in Mueller Hinton Broth were added to 100 μL of serially diluted extracts (250 to 0.122 μg mL^−1^). Wells containing bacterial inoculum without extracts (growth control) and wells containing only broth and ethanol (background control) were included in each plate. Controls were set up with ethanol in amounts corresponding to the highest quantity present in the test solution where appropriate. The experiments were replicated three times on different occasions with triplicate samples analyzed per replicate, and the antibacterial standard gentamycin was co-assayed under the same conditions. The microplates were incubated at 37 °C for 24 h, and the bacterial cell growth was assessed by measuring the optical density of cultures at 600 nm at zero (OD_1_) and 24 h (OD_2_) with a Tecan Spark Multimode Microplate Reader (Tecan Trading AG, Switzerland). The MIC was defined as the lowest concentrations of compound at which there was no visual turbidity due to microbial growth. Growth inhibition was estimated by the following formula:

Growth Inhibition (%) = ((OD_control_ − OD_corr_)/OD_control_) × 100

where OD_control_ is growth control at 24 h and OD_corr_ is OD_2_ − OD_1_.

### 2.8. Statistical Data Processing

The normality of the distribution of numeric variables was tested by the Shapiro–Wilk test. Since data do not follow the normal distribution, the comparison of sage extracts with the concentration of carnosic acid and carnosol and antibacterial activity was performed using the nonparametric Spearman coefficient of correlation. Data obtained from this study were processed in the STATISTICA 12.0 statistical program (Statsoft, Inc., Tulsa, OK, USA). All tests were performed at a level of significance of α = 0.05.

## 3. Results and Discussion

Process parameters for SFE were determined by BBD and are tabulated in Table 1. These parameters are used for the evaluation of extraction possibilities of carnosic acid and carnosol from sage leaves. In our previous work [42], where we performed SFE of sage leaves, we targeted the extraction of different volatile compounds, applying a range of process parameters, pressures of 10–30 MPa, temperatures of 40–60 °C, and CO_2_ flow rates of between 1–3 kg h^−1^ for 90 min, and the particle size of the plant material was 0.478 ± 0.36 mm. The same conditions were applied in this research as well, however we focused on the content of carnosic acid and carnosol in the obtained extracts, as prominent antioxidants in sage.

Process parameters of extraction experiments are tabulated in Table 2. The extraction process was optimized using RSM in order to achieve the highest amount of targeted compounds. The content of carnosic acid in sage extracts varied between 0.290–120.0 µg mg^−1^ of extract, depending on the applied extraction parameters. The lowest yield of carnosic acid was obtained at a pressure of 10 MPa and temperature of 50 °C, while the highest yield was obtained at 20 MPa and 40 °C (Table 2). The content of carnosol varied depending on the parameters used in the range of 0.460–65.5 µg mg^−1^, with the lowest yield obtained at 10 MPa and 50 °C and the highest yield at 20 MPa and 50 °C.

**Figure 1:**
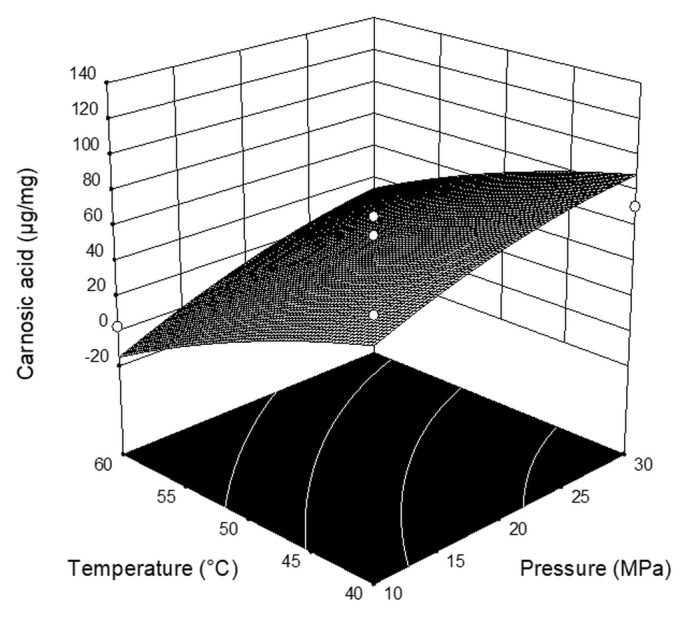
Three-dimensional plots for the obtained content of carnosic acid as a function of the extraction pressure and temperature.

As is evident from Figure 1 (response surface plots for carnosic acid) and Table 3 (analysis of variance, ANOVA), pressure, temperature, and CO_2_ flow rate statistically significantly influenced the content of carnosic acid (*p* = 0.0063; *p* = 0.0282, *p* = 0.0198) in the obtained extracts. The content of carnosic acid increased with the pressure and CO_2_ flow rate, while the increase of temperature decreased the content of carnosic acid. Interactions between extraction parameters (*p* > 0.05) did not show a significant influence on the extract carnosic acid composition.

**Figure 2:**
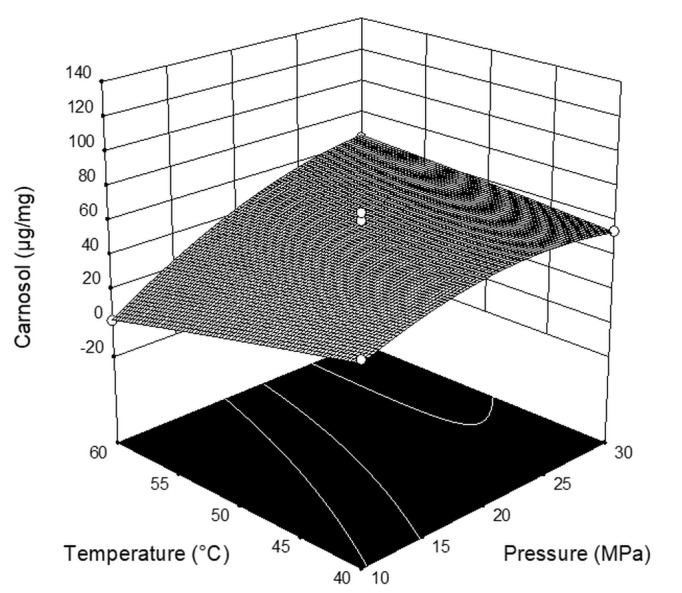
Three-dimensional plots for the obtained content of carnosol as a function of the extraction pressure and temperature.

Figure 2 (response surface plots for carnosol) and Table 3 (ANOVA data) demonstrate that the content of carnosol was significantly affected by the pressure (*p* = 0.0014), i.e., the content of carnosol increased with the increase in pressure. Unlike the effect on carnosic acid, neither temperature (*p* = 0.1360), CO_2_ flow rate (*p* = 0.1258), nor interaction between parameters showed significant statistical influence on the content of carnosol.

According to previous research, the amounts of antioxidant components in plant extracts are determined by the type of extraction and solvent used [50]. It is well known that carnosic acid degrades rapidly in methanol [15,51], and that in extracts with petroleum ether a small amount of carnosic acid is extracted [50]. In methanolic leaf extract of *S*. *officinalis*, the amounts of carnosic acid and carnosol were quantified as 14.6 mg g^−1^ DW and 0.4 mg g^−1^ DW, respectively [16]. The concentrations of carnosol and carnosic acid in 100 mL of aqueous infusion of sage were 0.66 ± 0.19 mg and 1.31 ± 0.33 mg [52], respectively, while in acetone they were 1.66 ± 0.21 mg g^−1^ and 12.40 ± 0.43 mg g^−1^, respectively [53]. In methanolic extracts of 12 samples of *S. officinalis* L. from Northern Italy, the content of carnosic acid was 0.2–7.1 g kg^−1^ of extract, while the content of carnosol was 1.1–9.0 g kg^−1^ of extract. In methanolic extracts of sage from Tunisia, the content of carnosic acid was 746–3110 µg g^−1^ of dry plant material weight, and depended on geographical location [54]. Additionally, supercritical fluid extraction has been used in plant material extraction considering that it can be performed at low temperatures in a short time, which effectively prevents the oxidation of carnosic acid during extraction and can provide clean extracts without residual of solvent [38,55,56]. 

As already stated, the data describing the optimal conditions for the extraction of carnosic acid and carnosol from sage using SFE are not available in the literature. However, Caldera et al. [57] investigated the SFE of carnosic acid and carnosol from rosemary. They concluded that the interaction between extraction temperature and time exhibited the most significant influence on the content of carnosic acid, while the extraction temperature and extraction time exhibited the most significant influence on the content of carnosol. Our results differ from those published, which is understandable since we were investigating a different plant material considering different extraction parameters; they can be explained if we consider the solubility of carnosic acid in CO_2_. The solubility of carnosic acid in SFE with ethanol as a co-solvent was investigated by Cháfer et al. [58]. The solubility of carnosic acid increases with the pressure and the amount of ethanol, while, in the range of pressures that they have explored, the solubility of carnosic acid is higher at lower temperatures, which is consistent with our results. These data on the solubility of the certain components during the extraction process using different parameters are good evidence that each medicinal plant behaves differently during extraction and that each active component is extracted differently depending on the raw material from which it is extracted.

Therefore, the optimization of the extraction process is necessary, and it can be achieved using BBD or some other design. Based on the BBD, the estimated coefficients of second-order response models for carnosol and carnosic acid in *S. officinalis* extracts are given in Table 4. The *R*^2^ was 0.861 and 0.901 for carnosic acid and carnosol, respectively, which indicates that the empirical model shows a good fit with empirical data (*R*^2^ are close to 1). According to the ANOVA results (Table 4), the models for both investigated responses (the content of carnosic acid and carnosol in sage extracts) were statistically significant (*p* ≤ 0.05), and the error analysis that showed a non-significant lack of fit (*p* = 0.0874–0.8893). Therefore, the influence of the SFE parameters we applied on carnosol and carnosic acid content can be described by a second-order polynomial model. 

According to RSM, the following optimization conditions were proposed for calculations: the maximum extraction yield as well as maximum content of carnosic acid and carnosol in obtained extracts. The extraction yields mentioned above were taken from previous work [42]. By applying the desirability function method [59], the optimum extraction conditions were obtained at a pressure of 29.5 MPa, a temperature of 49.2 °C, and a CO_2_ flow rate of 3 kg h^−1^. Under these optimal conditions, the yield of sage extract was calculated to be 6.54%, the carnosic acid content to be 105 µg mg^−1^, and the carnosol content to be 56.3 µg mg^−1^, which is in very close agreement with obtained experimental data (run 6, Table 2). The desirability for this optimization was 0.874. 

The quantitative evaluation of total phenolics in supercritical fluid sage leaf extracts as estimated by the method of Folin–Ciocalteu revealed that *S. officinalis* exhibited high and variable contents ranging from 1.02 to 9.15 mg of GAE g^−1^ of DM (Table 5). The highest total phenolic content (TPC) (9.15 ± 0.09 mg of GAE g^−1^ of DM) was recorded in sage extract obtained at 30 MPa and 40 °C with 2 kg h^−1^ CO_2_ flow rate with a carnosic acid content of 71.94 µg mg^−1^ and a carnosol content of 54.75 µg mg^−1^, whereas the lowest content (1.02 ± 0.02 mg of GAE g^−1^ of DM) was found in the case of sage extract obtained at 30 MPa and 60 °C with 2 kg h^−1^ CO_2_ flow rate with a carnosic acid content of 18.9 µg mg^−1^ and a carnosol content of 64.2 µg mg^−1^. The antioxidant activities of the supercritical fluid sage leaf extracts at a concentration of 25 µg mL^−1^ were evaluated by the scavenging activities of DPPH, as shown in Table 5. Sage extract obtained at 30 MPa and 40 °C with 2 kg h^−1^ CO_2_ flow rate with a carnosic acid content of 71.9 µg mg^−1^ and a carnosol content of 54.8 µg mg^−1^ also exhibited the highest antioxidant activity (80.0 ± 0.68%) amongst the investigated supercritical extracts, whereas the lowest antioxidant activity (26.9 ± 0.91%) was found in the case of sage extract obtained at 30 MPa and 60 °C with 2 kg h^−1^ CO_2_ flow rate with a carnosic acid content of 18.9 µg mg^−1^ and a carnosol content of 64.2 µg mg^−1^. Non-parametric Spearman correlation analysis of all data points demonstrated significant (*p* < 0.050) strong positive correlation between the carnosic acid content and TPC results (*R* = 0.7909), and significant (*p* < 0.050) moderate positive correlation between the carnosic acid content and radical scavenging activity (*R* = 0.5636).

Supercritical fluid extracts from sage leaves were tested for in vitro antibacterial activity against *E. coli*, *P. aeruginosa, B. subtilis*, and *S. aureus.* MIC and IC_50_ results are shown in Table 6. 

As shown in Table 6, all tested extracts showed good antibacterial activities against *E. coli*, *P. aeruginosa, B. subtilis*, and *S. aureus.* Extracts were more active against gram-positive bacteria than gram-negative bacteria. The main reason for the differences in bacterial susceptibility could be the outer membrane surrounding the cell wall in gram-negative bacteria, which restricts the diffusion of compounds through its lipopolysaccharide covering, as previously reported [60]. The best antibacterial activity was seen against *B. subtilis* and the lowest activity against *E. coli*. As reported, carnosic acid, carnosol, rosmanol, and ferruginol are also responsible for the biological activity of sage (*Salvia* sp.) along with the phenolic rosmarinic and salvianolic acids [61]. The results in Table 6 show supercritical fluid sage extracts to be very effective. Obviously, MIC values are in accordance with IC_50_ however are not as accurate. Among them, as shown in Table 6, the extract with a carnosic acid content of 116 µg mg^−1^ and a carnosol content of 60.6 µg mg^−1^ showed the lowest IC_50_ 10.82 ± 0.02 µg mL^−1^ against *B. subtilis*, and the extract with a carnosic acid content of 66.2 µg mg^−1^ and a carnosol content of 61.02 µg mg^−1^ showed the highest IC_50_ 40.4 ± 0.19 µg mL^−1^ against *E. coli.* As shown in Table 7, all the extracts revealed excellent growth inhibition of all tested bacteria at 62.5 µg mL^−1^ extract concentration, while at the extract concentration of 15.6 µg mL^−1^ growth inhibition varied from 14.5–76.8%. Among them, the extract with a carnosic acid content of 116 µg mg^−1^ and a carnosol content of 60.6 µg mg^−1^ (run 6, Table 2) showed the best antibacterial activity, especially against *B. subtilis*, with inhibition rates of 98.33 ± 1.19% at a concentration of 62.5 µg mL^−1^ and 76.79 ± 0.88% at a concentration of 15.6 µg mL^−1^. According to the results obtained in the present work, the carnosic acid/carnosol ratio of the sage extracts seems to affect the antibacterial activity of the extracts. 

As shown in Table 6 and Table 7, a higher content of carnosic acid in relation to carnosol showed better antibacterial activities of the supercritical fluid sage extracts. We can not claim that the higher content of carnosic acid improves antibacterial activity, since SFE extracts contained carnosic acid and carnosol, as well as many other components such as oxygenated monoterpenes, α-humulenes, viridiflorol, and manool. Our findings are in agreement with those of different authors, such as Klancnik et al. [62] and Bubonja-Sonje et al. [63], who found that the biological activities of rosemary extracts are directly related to the presence of carnosic acid as the major phenolic component, but contrast with the findings of Jordán et al. [64], who found that a higher concentration of carnosol in relation to carnosic acid with same rosmarinic acid content improves the antibacterial activities of methanolic rosemary extracts against *Listeria monocytogenes* and *Staphylococcus aureus* strains.

## 4. Conclusions

In this work, optimization of the extraction of carnosol and carnosic acid from sage leaves using SFE was performed. Only the pressure significantly affected the extraction of carnosol, while in the case of carnosic acid, all investigated parameters—pressure, temperature, and CO_2_ flow rate—showed a significant effect. The results revealed that the optimal conditions for the maximum extraction of carnosic acid and carnosol were at a pressure of 29.45 MPa, a temperature of 49.19 °C, and a CO_2_ flow rate of 3 kg h^−1^. It was observed that by changing the applied pressure and temperature it is possible to obtain an extract with completely different contents of the desired components. The highest total phenolic content (TPC) (9.15 ± 0.09 mg of GAE g^−1^ of DM) and highest antioxidant activity (79.98 ± 0.68%) at 25 µg mL^−1^ concentration amongst the investigated supercritical fluid sage extracts was recorded in extract obtained at 30 MPa and 40 °C with 2 kg h^−1^ CO_2_ flow rate with a carnosic acid content of 71.94 µg mg^−1^ and a carnosol content of 54.75 µg mg^−1^. In this study, the best antibacterial efficiency was confirmed for supercritical fluid sage extract formulations with higher carnosic acid content against all the tested strains, especially gram-positive *B. subtilis*. Gram-negative tested strains were less susceptible, which could be related to the lower permeability of their surface for phenolic compounds.

## Figures and Tables

**Table 1:** Coded and real levels of independent variables for the designed experiment.

Independent Variable	Symbol	Level		
		Low (−1)	Middle (0)	High (+1)
Pressure (MPa)	*X*_1_	10	20	30
Temperature (°C)	*X*_2_	40	50	60
CO_2_ flow rate (kg h^−1^)	*X*_3_	1	2	3

**Table 2:** Experimental matrix and values of observed response.

Run	Pressure (MPa)	Temperature (°C)	CO_2_ Flow Rate (kg h^−1^)	Extraction Yield * (%)	Carnosic Acid (µg mg^−1^_extract_)	Carnosol (µg mg^−1^_extract_)
1	10	40	2	0.659	67.94	40.71
2	20	40	1	3.385	48.04	61.70
3	20	40	3	4.026	120.05	51.08
4	10	50	3	1.144	25.49	39.77
5	20	50	2	3.768	47.56	65.51
6	30	50	3	7.361	116.25	60.63
7	30	50	1	5.238	61.78	61.47
8	20	60	3	5.477	38.53	58.00
9	10	50	1	0.242	0.29	0.46
10	10	60	2	0.365	2.93	1.80
11	20	50	2	3.305	39.78	37.28
12	30	60	2	4.316	18.93	64.19
13	20	50	2	2.552	55.61	46.72
14	30	40	2	3.803	71.94	54.75
15	20	60	1	4.891	1.64	35.19
16	20	50	2	4.528	66.20	61.02

* Data are from Jokić et al. (2018).

**Table 3:** Regression coefficient of polynomial function of all response surfaces.

Term	Coefficients	Standard Error	*F*-Value	*p*-Value *
**Carnosic acid**				
Intercept	52.99	10.58		
*X*_1_	21.53	7.48	8.28	0.0282
*X*_2_	−30.74	7.48	16.87	0.0063
*X*_3_	23.57	7.48	9.92	0.0198
*X*_1_^2^	−6.48	10.58	0.38	0.5628
*X*_2_^2^	−5.37	10.58	0.26	0.6298
*X*_3_^2^	−5.15	10.58	0.24	0.6439
*X*_1_ *X*_2_	3.00	10.58	0.080	0.7865
*X*_1_ *X*_3_	7.32	10.58	0.48	0.5153
*X*_2_ *X*_3_	−8.78	10.58	0.69	0.4385
*R*^2^ = 0.8611				
**Carnosol**				
Intercept	52.63	5.04		
*X*_1_	19.79	3.56	30.85	0.0014
*X*_2_	−6.13	3.56	2.96	0.1360
*X*_3_	6.33	3.56	3.16	0.1258
*X*_1_^2^	−11.59	5.04	5.29	0.0534
*X*_2_^2^	−0.68	5.04	0.018	0.0934
*X*_3_^2^	−0.46	5.04	0.03	0.1481
*X*_1_ *X*_2_	12.09	5.04	5.75	0.0611
*X*_1_ *X*_3_	−10.04	5.04	3.97	0.8973
*X*_2_ *X*_3_	−0.46	5.04	2.75	0.9300
*R*^2^ = 0.9013				

* *p* < 0.01 highly significant; 0.01 ≤ *p* < 0.05 significant; *p* ≥ 0.05 not significant.

**Table 4:** Analysis of variance (ANOVA) of the modeled responses.

Source	Sum of Squares	Degrees of Freedom	Mean Square	*F*-Value	*p*-Value *
*Carnosic acid*					
*The recovery*					
Model	16663.23	9	1851.47	4.13	0.0491
Residual	2688.70	6	448.12		
Lack of fit	2305.19	3	768.40	6.01	0.0874
Pure error	383.51	3	127.84		
Total	19351.94	15			
*Carnosol*					
*The recovery*					
Model	5560.32	9	617.81	6.09	0.0198
Residual	609.14	6	101.52		
Lack of fit	102.25	3	34.08	0.20	0.8893
Pure error	506.89	3	168.96		
Total	6169.46	15			

* *p* < 0.01 highly significant; 0.01 ≤ *p* < 0.05 significant; *p* ≥ 0.05 not significant.

**Table 5:** Total phenolics content (TPC) in supercritical fluid sage leaf extracts expressed as mg of GAE g^−1^ of DM and 2,2-diphenyl-1-picrylhydrazyl (DPPH) radical scavenging activity expressed as % DPPH radical scavenging activity at 25 µg mL^−1^ concentration.

Run	Total Phenolics Content (TPC) (mg_GAE_ g_DM_^−1^)	DPPH Radical Scavenging Activity (%)
1	2.79 ± 0.06	57.71 ± 0.33
2	3.08 ± 0.05	38.99 ± 0.32
3	ND	ND
4	2.26 ± 0.01	46.87 ± 3.60
5	3.97 ± 0.13	53.53 ± 3.37
6	4.32 ± 0.03	47.05 ± 1.77
7	ND	ND
8	ND	ND
9	ND	ND
10	ND	ND
11	2.72 ± 0.07	56.72 ± 0.65
12	1.02 ± 0.02	26.91 ± 0.91
13	3.17 ± 0.01	48.01 ± 0.86
14	9.15 ± 0.09	79.98 ± 0.68
15	2.65 ± 0.07	42.62 ± 1.69
16	2.96 ± 0.01	50.54 ± 1.98

ND: not determined. Data expressed as mean ± S.D.

**Table 6:** Comparison of minimum inhibitory concentrations (MIC) and 50% Growth Reduction (IC_50_) of supercritical fluid sage leaf extracts against *Escherichia coli*, *Pseudomonas aeruginosa, Bacillus subtilis*, and *Staphylococcus aureus* (µg mL^−1^).

Run	MIC (µg mL^−1^)				IC_50_ (µg mL^−1^)			
	*E. coli*	*P. aeruginosa*	*B. subtilis*	*S. aureus*	*E. coli*	*P. aeruginosa*	*B. subtilis*	*S. aureus*
1	31.25	31.25	15.625	31.25	29.53 ± 0.16	28.54 ± 0.05	12.45 ± 0.08	17.17 ± 0.09
2	31.25	31.25	15.625	31.25	27.89 ± 0.07	26.28 ± 1.76	12.69 ± 0.07	23.90 ± 0.10
3	ND	ND	ND	ND	ND	ND	ND	ND
4	31.25	31.25	15.625	15.625	27.03 ± 0.55	25.75 ± 0.52	13.97 ± 0.07	14.53 ± 0.04
5	31.25	31.25	15.625	31.25	31.18 ± 0.05	16.33 ±0.26	12.77 ± 0.06	22.75 ± 0.13
6	31.25	31.25	15.625	15.625	31.08 ± 0.08	17.82 ± 0.39	10.82 ± 0.02	14.61 ± 0.08
7	ND	ND	ND	ND	ND	ND	ND	ND
8	ND	ND	ND	ND	ND	ND	ND	ND
9	ND	ND	ND	ND	ND	ND	ND	ND
10	ND	ND	ND	ND	ND	ND	ND	ND
11	31.25	31.25	15.625	15.625	28.43 ± 0.36	22.23 ± 0.61	12.70 ± 0.08	14.10 ± 0.10
12	62.50	31.25	15.625	15.625	39.68 ± 0.21	22.36 ± 0.79	14.02 ± 0.16	14.12 ± 0.18
13	62.50	31.25	15.625	15.625	37.80 ± 0.83	23.78 ± 0.44	13.18 ± 0.08	12.46 ± 0.10
14	31.25	31.25	15.625	15.625	24.85 ± 0.08	23.57 ± 1.21	12.51 ± 0.11	15.04 ± 0.13
15	31.25	31.25	15.625	15.625	20.29 ± 0.62	27.98 ± 1.24	13.01 ± 0.16	13.75 ± 0.04
16	62.50	31.25	15.625	15.625	40.44 ± 0.19	23.61 ± 0.53	13.43 ± 0.04	17.91 ± 0.67
G	0.976	0.976	1.953	3.906	0.58 ± 0.10	0.91 ± 0.07	1.83 ± 0.01	3.06 ± 0.06

ND: not determined. G-gentamicin data expressed as mean ± S.D.

**Table 7:** Bacterial growth inhibition in the presence of supercritical fluid sage leaf extracts against *Escherichia coli*, *Pseudomonas aeruginosa, Bacillus subtilis*, and *Staphylococcus aureus*.

Run	Growth Inhibition (%)							
	*E. coli*		*P. aeruginosa*		*B. subtilis*		*S. aureus*	
	62.5 µg mL^−1^	15.625 µg mL^−1^	62.5 µg mL^−1^	15.625 µg mL^−1^	62.5 µg mL^−1^	15.625 µg mL^−1^	62.5 µg mL^−1^	15.625 µg mL^−1^
1	98.34 ± 0.35	16.73 ± 0.51	96.62 ± 1.84	31.93 ± 3.22	98.84 ± 0.29	72.19 ± 0.22	98.32 ± 0.06	46.33 ± 0.15
2	93.04 ± 0.19	20.10 ± 0.59	91.42 ± 1.56	35.39 ± 0.57	98.26 ± 0.44	71.80 ± 0.83	97.56 ± 0.22	21.97 ± 1.17
3	ND	ND	ND	ND	ND	ND	ND	ND
4	97.17 ± 0.28	14.45 ± 1.18	97.36 ± 0.30	38.30 ± 0.79	99.48 ± 010	61.62 ± 0.43	98.31 ± 0.12	57.57 ± 0.36
5	95.82 ± 0.28	23.91 ± 0.34	93.30 ± 0.65	49.41 ± 0.22	97.39 ± 0.42	68.05 ± 0.74	96.57 ± 0.87	32.24 ± 0.16
6	99.05 ± 1.19	39.22 ± 0.06	95.91 ± 0.96	49.63 ± 0.08	98.33 ± 0.38	76.79 ± 0.88	97.80 ± 0.57	55.47 ± 0.77
7	ND	ND	ND	ND	ND	ND	ND	ND
8	ND	ND	ND	ND	ND	ND	ND	ND
9	ND	ND	ND	ND	ND	ND	ND	ND
10	ND	ND	ND	ND	ND	ND	ND	ND
11	95.75 ± 1.62	28.04 ± 1.18	95.07 ± 1.29	44.10 ± 1.02	98.88 ± 0.22	72.31 ± 0.39	97.74 ± 0.19	56.69 ± 0.59
12	94.96 ± 0.35	20.21 ± 3.00	97.78 ± 1.37	31.93 ± 3.22	99.00 ± 0.22	60.99 ± 1.31	97.98 ± 0.09	60.77 ± 1.56
13	97.41 ± 0.82	26.80 ± 3.62	98.07 ± 0.41	41.50 ± 3.88	98.72 ± 0.22	67.30 ± 0.10	97.37 ± 0.66	74.93 ± 0.30
14	97.03 ± 0.24	37.76 ± 0.25	99.15 ± 0.15	39.71 ± 2.95	99.28 ± 0.18	71.42 ± 0.64	97.55 ± 0.18	52.30 ± 0.60
15	97.47 ± 1.10	40.72 ± 1.30	97.45 ± 0.70	42.32 ± 2.04	99.41 ± 0.45	69.85 ± 0.65	98.24 ± 0.29	61.11 ± 0.29
16	96.77 ± 0.41	28.47 ± 3.18	97.26 ± 2.11	40.38 ± 1.19	98.81 ± 0.11	65.04 ± 0.58	97.00 ± 0.86	46.29 ± 1.28
G	98.18 ± 0.76	95.00 ± 0.17	99.83 ± 0.02	96.83 ± 0.02	99.86 ± 0.14	94.83 ± 0.71	98.58 ± 0.56	97.00 ± 0.67

ND: not determined. G-gentamicin data expressed as mean ± S.D.
